# Supplementary material for: Serum microRNA miR-491-5p/miR-206 Is Correlated with Poor Outcomes/Spontaneous Hemorrhagic Transformation after Ischemic Stroke: A Case Control Study
Source: Brain Sci. 2022 Jul 28;12(8):999. doi: 10.3390/brainsci12080999 (PMC9405583; doi:10.3390/brainsci12080999)
Supplement: Supplementary file 1 [file brainsci-12-00999-s001.zip › brainsci-1815393-supplementary.pdf]

Table S1. The baseline characteristics of AIS patients with or without spontaneous HT.

		Overall（*n*=215）	HT（*n*=15）	Non HT（*n*=200）	*p*-value
**Age,years, (mean (SD))**		66.69 (14.52)	78.40 (9.27)	65.81 (14.47)	**0.001***
**Female, *n* (%)**		83 (38.6)	10 (66.7)	73 (36.5)	**0.041***
Onset to admission,h, median [IQR]		24.00 [8.50, 48.00]	24.00 [10.75, 36.00]	24.00 [8.75, 48.00]	0.563
Onset to blood sampling, h, median [IQR]		25.68 [5.75, 43.08]	19.76 [6.47, 28.48]	26.06 [5.73, 43.84]	0.314
**GCS on admission,median [IQR]**		15.00 [13.00, 15.00]	12.00 [10.00, 14.00]	15.00 [13.00, 15.00]	**0.005***
**NIHSS on admission,median [IQR]**		6.00 [3.00, 12.00]	13.00 [6.50, 15.50]	6.00 [3.00, 12.00]	**0.047***
Axillary temperature,℃, mean(SD)		36.53(0.44)	36.69(0.14)	36.51(0.03)	0.109
History of risk factors					
Hypertension, *n* (%)		124 (57.7)	9 (60.0)	115 (57.5)	1
DM, *n* (%)		53 (24.7)	3 (20.0)	50 (25.0)	0.902
Hyperlipodermia, *n* (%)		8 (3.7)	1 (6.7)	7 (3.5)	1
**AF, *n* (%)**		31 (14.4)	7 (46.7)	24 (12.0)	**0.001***
Acute heart infarction, *n* (%)		4 (1.9)	0 (0.0)	4 (2.0)	1
Valvular heart disease, *n* (%)		11 (5.1)	0 (0.0)	11 (5.5)	0.745
Transient ischemic attack, *n* (%)		6 (2.8)	1 (6.7)	5 (2.5)	0.895
Acute ischemic stroke, *n* (%)		43 (20.0)	3 (20.0)	40 (20.0)	1
Hemorrhagic stroke, *n* (%)		3 (1.4)	0 (0.0)	3 (1.5)	1
Therapies before admission					
Antiplatelet therapy, *n* (%)		28 (13.0)	1 (6.7)	27 (13.5)	0.718
Lipid lowering, *n*(%)		19 (8.8)	1 (6.7)	18 (9.0)	1
Anticoagulant therapy, *n*(%)		10 (4.7)	0 (0.0)	10 (5.0)	0.802
**TOAST classification (%)**	LAA	62 (28.8)	0 (0.0)	62 (31.0)	**0.001***
	SAO	49 (22.8)	1 (6.7)	48 (24.0)	
	CE	58 (27.0)	11 (73.3)	47 (23.5)	
	SOE	5 (2.3)	0 (0.0)	5 (2.5)	
	SOU	41 (19.1)	3 (20.0)	38 (19.0)	
**ECASS classification (%)**	No	200 (93.0)	0 (0.0)	200 (100.0)	**<0.001***
	HI1	6 (2.8)	6 (40.0)	0 (0.0)	
	HI2	5 (2.3)	5 (33.3)	0 (0.0)	
	PH1	4 (1.9)	4 (26.7)	0 (0.0)	
	PH2	0 (0.0)	0 (0.0)	0 (0.0)	
Levels of miRNA expression, fold difference					
miR-21-5p, median [IQR]		0.82 [0.18, 4.10]	0.73 [0.33, 3.15]	0.83 [0.17, 4.07]	0.858
miR-491-5p, median [IQR])		0.98 [0.29, 2.45]	1.07 [0.35, 3.41]	0.98 [0.29, 2.45]	0.778
miR-3123, median [IQR]		1.60 [0.08, 12.26]	0.88 [0.01, 16.57]	1.81 [0.12, 11.69]	0.364

miR-206, median [IQR]	1.15 [0.29, 7.57]	7.55 [1.35, 50.79]	0.99 [0.28, 5.93]	**0.04***

AIS, acute ischemic stroke; HT, hemorrhagic transformation; SD, standard deviation; IQR, interquartile range; GCS, Glasgow Coma Scale; NIHSS, National Institutes of Health Stroke Scale; DM, diabetes mellitus; AF, atrial fibrillation; TOAST, Trial of Org 10,172 in Acute Stroke Treatment; LAA, large-artery atherosclerosis; SAO, small-artery occlusion; CE, cardioembolism; SOE, acute stroke of other determined etiology; SUE, stroke of underdetermined etiology; ECASS, The European Cooperative Acute Stroke Study; HI, hemorrhagic infarction; PH, parenchymal hemorrhage

*$p < 0.05$
